# Recent Advances in Poly(vinyl alcohol)-Based Hydrogels

**DOI:** 10.3390/polym16142021

**Published:** 2024-07-15

**Authors:** Maria Bercea

**Affiliations:** “Petru Poni” Institute of Macromolecular Chemistry, 41-A Grigore Ghica Voda Alley, 700487 Iasi, Romania; bercea@icmpp.ro

**Keywords:** poly(vinyl alcohol), hydrogel, wound dressings, tissue engineering, strain sensors

## Abstract

Poly(vinyl alcohol) (PVA) is a versatile synthetic polymer, used for the design of hydrogels, porous membranes and films. Its solubility in water, film- and hydrogel-forming capabilities, non-toxicity, crystallinity and excellent mechanical properties, chemical inertness and stability towards biological fluids, superior oxygen and gas barrier properties, good printability and availability (relatively low production cost) are the main aspects that make PVA suitable for a variety of applications, from biomedical and pharmaceutical uses to sensing devices, packaging materials or wastewater treatment. However, pure PVA materials present low stability in water, limited flexibility and poor biocompatibility and biodegradability, which restrict its use alone in various applications. PVA mixed with other synthetic polymers or biomolecules (polysaccharides, proteins, peptides, amino acids etc.), as well as with inorganic/organic compounds, generates a wide variety of materials in which PVA’s shortcomings are considerably improved, and new functionalities are obtained. Also, PVA’s chemical transformation brings new features and opens the door for new and unexpected uses. The present review is focused on recent advances in PVA-based hydrogels.

## 1. Introduction

**Figure 1:**
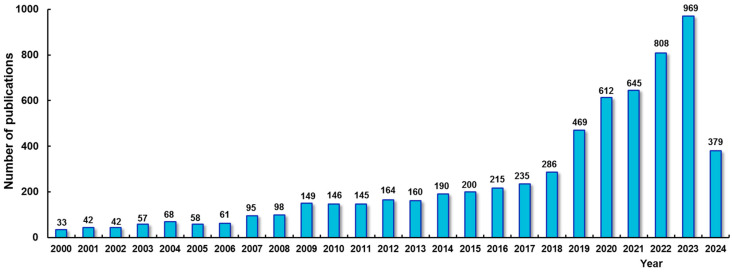
The frequency of publications identified on the Web of Science database [1] during the period 2000–2024, using the keywords “PVA hydrogel”.

Poly(vinyl alcohol) (PVA) is one of the most important water-soluble synthetic polymers, being inexpensive, non-toxic, readily biodegradable and able to form environment-friendly hydrogels, films and membranes. During the last few years, significant advances were registered in the design of PVA-based materials from new and improved preparation and characterization procedures to testing methods for various and possible unique uses. The high interest for PVA-based hydrogels is reflected by a continuously increasing number of original papers and reviews reported during the last few years (Figure 1).

A large number of PVA-based hydrogels are suitable for biomedical and engineering applications, tissue engineering scaffolds, drug delivery, cell culture, implanted artificial muscles and organs, sensors, wound dressings, soft robotics, food packaging or environmental applications. The properties and functions required for a particular application can be fine-tuned by a careful selection of the crosslinking method, PVA characteristics and of other used components for preparing high-performance composites [2,3,4,5,6,7,8,9,10].

This review is focused mainly on the trends regarding PVA hydrogels as presented in recent studies and some perspectives for future research.

## 2. Structure and Properties of PVA

**Scheme 1:**
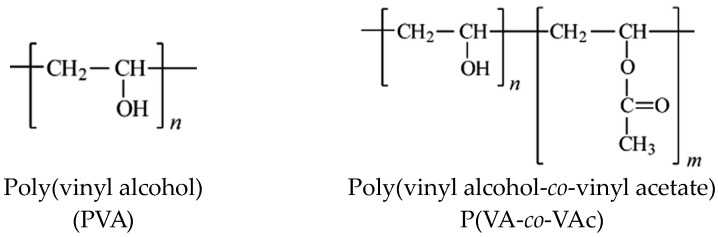
The chemical structure of poly(vinyl alcohol) and poly(vinyl alcohol-*co*-vinyl acetate). The commercial PVA samples have *n* >> *m*.

PVA is produced at an industrial scale through the hydrolysis of poly(vinyl acetate); thus, different degrees of hydrolysis (DHs) (also denoted as the degree of saponification) can be achieved. The corresponding monomer, vinyl alcohol, is thermodynamically unstable and spontaneously transforms into its enol form of acetaldehyde, so the direct polymerization of vinyl alcohol is not possible. PVA samples of different DHs and molecular weights are available on the market [11]. In terms of DHs, PVA samples can be classified as fully hydrolyzed (DH ≥ 97.5%) or partially hydrolyzed (DH < 97.5%), the last category being a copolymer structure, poly(vinyl alcohol-*co*-vinyl acetate) [12] (Scheme 1); these characteristics influence the overal properties [13]. Thus, the polymer’s solubility in water improves with increasing DH only up to DH = 91% [12]. Fully hydrolyzed PVA samples present good stability at room temperature and are dissolved by water and its mixtures with organic solvents, such as dimethyl sulfoxide (DMSO), dimethylformamide (DMF) or N-methyl-2-pyrrolidone (NMP), by heating at high temperatures (above 80 °C) for several tens of minutes when the strong intermolecular hydrogen bonds are broken [13,14,15]. Water behaves as a marginal solvent for PVA; in aqueous solution, A_2_ is about 1.4 × 10^−4^ mol/(cm^3^g^2^), whereas NMP or DMSO appear as good solvents, A_2_ > 2 × 10^−3^ mol/(cm^3^g^2^) when the polymer coil exhibits an extended conformation [16]. Due to the presence of hydrophobic acetate groups, partially hydrolyzed PVA chains are less soluble in polar solvents, when, according to molecular dynamic simulation, the polymer/solvent’s thermodynamic affinity and the surface energy are lower [17]. The solution state (diluted, semidiluted, concentrated, entangled or non-entangled) and intermolecular interactions depend on temperature, concentration and molecular weight [18,19] and dictate the viscosity and viscoelasticity of aqueous solutions, influencing the manufacturing of PVA hydrogels [18]. Below a critical reduced concentration value, (c × [η])_cr_ (where c × [η] is a dimensionless parameter defined as concentration × intrinsic viscosity), the network formation cannot take place; only isolated droplets are generated. The parameter (c × [η])_cr_ is about 2 for PVA in water [18] and indicates the macroscopic percolation transition limit. Above (c × [η])_cr_, the overlapping between the macromolecular coils determines a phase separation, when a polymer-rich phase is formed and gelation occurs [18,20]. The use of freshly prepared solutions is recommended for preparing hydrogels; otherwise, the prolonged storage of PVA solutions causes the appearance of aggregates; furthermore, several species of microorganisms can degrade the polymer [21]. The thermal and shear history influence the network formation [22,23,24] and mechanical properties of PVA-based materials [25,26].

The DH (or the content of acetate groups along PVA chains) is an important parameter which impacts the chemical properties [27,28] and formation of supramolecular structures [29] but also the structure and stability of PVA-based materials [23,30]. The DH and the interface characteristics of PVA influence its compatibility with other macromolecules [31,32,33], organic and inorganic materials [3,4,30,34] and additionally the printing ability [35] or electrospinning processes [36], film forming by solution casting [17] and mechanical behavior of materials [37]. The salting-out effects of kosmotropic anions or addition of organic chaotropes into aqueous PVA solutions reduces the polymer chains’ solvation and enhances their ability to generate intermolecular hydrogen bonds (H-bonds) [30,34,38]. It was shown that heavy metal compounds form complexes with PVA molecules [39].

For commercially available PVA samples, the values of the polymerization degree, *n*, are between 200 and 5000, corresponding to molecular weights in the range of 10^4^ g/mol to 2 × 10^5^ g/mol. PVA samples with high hydrolyzed grades (>96%) are used for preparing hydrogels. The tacticity of PVA chains depends on the method of synthesis. As an example, atactic PVA is obtained by the hydrolysis of poly(vinyl acetate), the highly isotactic structure results from the polymerization of vinyl *tert*-butyl ether in toluene [40] and the highly syndiotactic PVA sample is obtained through the polymerization of vinyl trimethylsilyl ether in nitroethane [41]. The tacticity of PVA chains influences the intermolecular interactions and the ability to form crystalline regions and to generate physical networks. The gel strength and mechanical properties of syndiotactic PVA are considerably higher as compared with atactic chains [42,43,44]. The structure and properties of PVA hydrogels are also influenced by processing parameters: solvent, temperature and concentration [18,45,46,47].

Nanofibers were obtained through electrospinning, using aqueous solutions of 7.5% PVA (mixtures of PVA samples with *n* = 1700 and *n* = 4000). A higher content of long PVA chains was required to ensure fiber continuity, superior crystallinity, mechanical properties and thermal stability [48].

The melting point of fully hydrolyzed PVA samples was found to be between 220 °C and 230 °C, while for partially hydrolyzed, it decreased to 180–190 °C; the glass transition temperature varied between 65 °C and 85 °C, and the decomposition occurred around 220–250 °C [49].

It was shown that PVA chains are susceptible to microbial degradation in the presence of suitable microorganisms [11,50]. The biodegradation of PVA macromolecular chains takes place from 1–2 weeks to 4 months in industrial or ocean aerobic/anaerobic conditions, in the presence of degrading microorganisms (bacteria, fungi etc.). The two-step biodegradation process includes firstly an enzymatic oxidation of –OH groups into mono- or diketones followed by hydrolysis [51,52]. PVA can be also recycled by using other treatments, such as photochemically initiated degradation, ultrasonic- or radiation-induced degradation, adsorption by various materials etc. [51]. Recently, a promising procedure was reported on for industrial applications involving a cascade of four steps using three enzymes and an in situ cofactor (NADP^+^ with formation of NADPH) for recycling PVA chains or PVA modified with succinic anhydride [53,54].

The development of materials with multifunctional characteristics is a permanent challenge. According to published studies, PVA is one of the most versatile polymers used for preparing various composites and nanomaterials in combination with other natural and synthetic polymers, inorganic/organic compounds and peptides/proteins [8,9,10,31,55,56,57,58,59,60,61,62,63]. The control of the molecular structure combined with polymer chemistry allows for the fine-tuning of sophisticated supramolecular organization and chain dynamics that define the macroscopic properties of PVA-based materials [13,64,65,66,67,68].

The network- and film-forming ability, superior mechanical properties, non-toxicity, partial biodegradability, good adhesion and processability are the most important characteristics that make PVA a suitable candidate in various applications. Given the growing interest in producing more competitive materials, some of the recent advances in PVA-based hydrogels are presented below.

## 3. Preparation Methods of PVA Hydrogels

Various strategies are currently used to physically or chemically connect PVA chains into a 3D porous network structure, in order to generate materials with tunable characteristics through a variety of interactions, from weak physical structural assemblies to strong covalent bonding. Thus, PVA hydrogels with different stiffness and porosity were produced by physical (freezing/thawing, directional freezing or salting-out methods, ultraviolet (UV) or gamma irradiation, annealing or heat treatment), chemical (use of chemical crosslinkers, copolymerization, modifications of OH group) or combined procedures [2,3,4,5,6,7,8,9,10,18,37,65].

Physically crosslinked PVA hydrogels have attracted particular attention because of their inherent lack of toxicity, high degree of swelling in water and tunable viscoelastic properties. The covalent bonds established in the presence of crosslinking agents induce superior mechanical properties, improved thermal stability or stability to solvents. However, crosslinking reactions can bring high toxicity, low degradability or undesirable reaction products, effects that are not desired in the production of biomaterials, biopackaging or pharmaceutical products. Thus, non-covalent bonding is often preferred as a more friendly procedure to engineer materials with non-toxic properties, and covalent approaches with natural crosslinking agents are continuously exploited. The insolubility, mechanical properties and thermal stability of chemically crosslinked PVA are of interest in producing membranes [27].

Hydrogel strength and swelling ability represent two of the most important characteristics, inversely correlated with one another, and they are considered as indicators of network performances. Stronger gels have a higher number of junction points in their structures, which increases the crosslinking density and reduces the amount of available space for water or other molecule absorption. Preparative methods are creatively used to provide multiple functions to PVA-based materials with minimum costs and efforts.

Various strategies have been applied to produce PVA-based hydrogels with different stiffness and porosity: physical (freezing/thawing, directional freezing or salting-out methods, ultraviolet (UV) or gamma irradiation, annealing or heat treatment), chemical (use of chemical crosslinkers, copolymerization, modifications of OH group) or combined procedures [2,3,4,5,6,7,8,9,10,18,37,65].

### 3.1. PVA Hydrogels Obtained by Repeated Freezing/Thawing Cycles Applied to Aqueous Solutions

**Figure 2:**
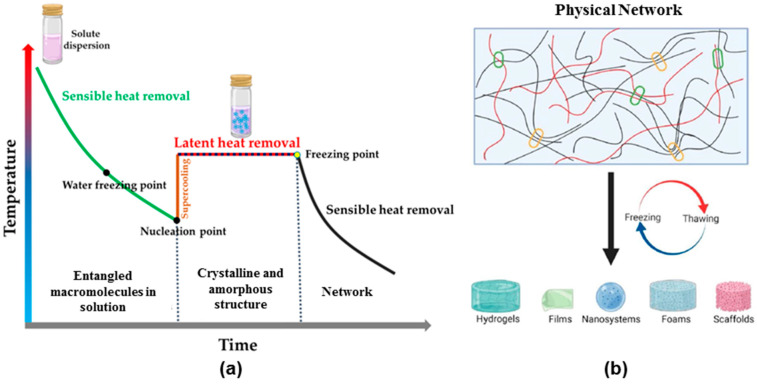
Schematical presentation of (**a**) temperature–time curve during FT process and (**b**) structure of PVA networks designed for different applications. Adapted with permission from [71], copyright 2024, BioMed Central Ltd., Part of Springer Nature.

It is now well known that physical networks can be generated by applying repeated freezing/thawing (FT) treatments to homogeneous aqueous PVA solutions [3,4,7,18,20,29,30,69,70,71,72,73,74,75,76,77,78,79,80]. This procedure involves supercooling, thawing (nucleation), the growth of crystalline zones and network aging (Figure 2) [71]. The phase separation of frozen water and formation of PVA crystalline domains during the FT process are the responsive factors of hydrogel formation. As the ice crystals develop during the freezing process, they push PVA chains towards one another, and thus, the PVA-rich phase segregation is accelerated [7,18,20,45,81].

Thus, the gelation was attributed to the formation of intermolecular H-bonds and crystalline zones in PVA-rich regions that act as physical crosslinking points generating a porous network structure. The degree of crystallinity induced by cryogenic treatments depends on solution preparation, the temperature–time history of the PVA system, the number of applied FT cycles, thawing temperature, cooling/heating rate or the presence of other molecules in the PVA solution. These factors influence the hydrogel’s crystallinity and porosity, mechanical properties, swelling behavior and viscoelastic properties.

**Figure 3:**
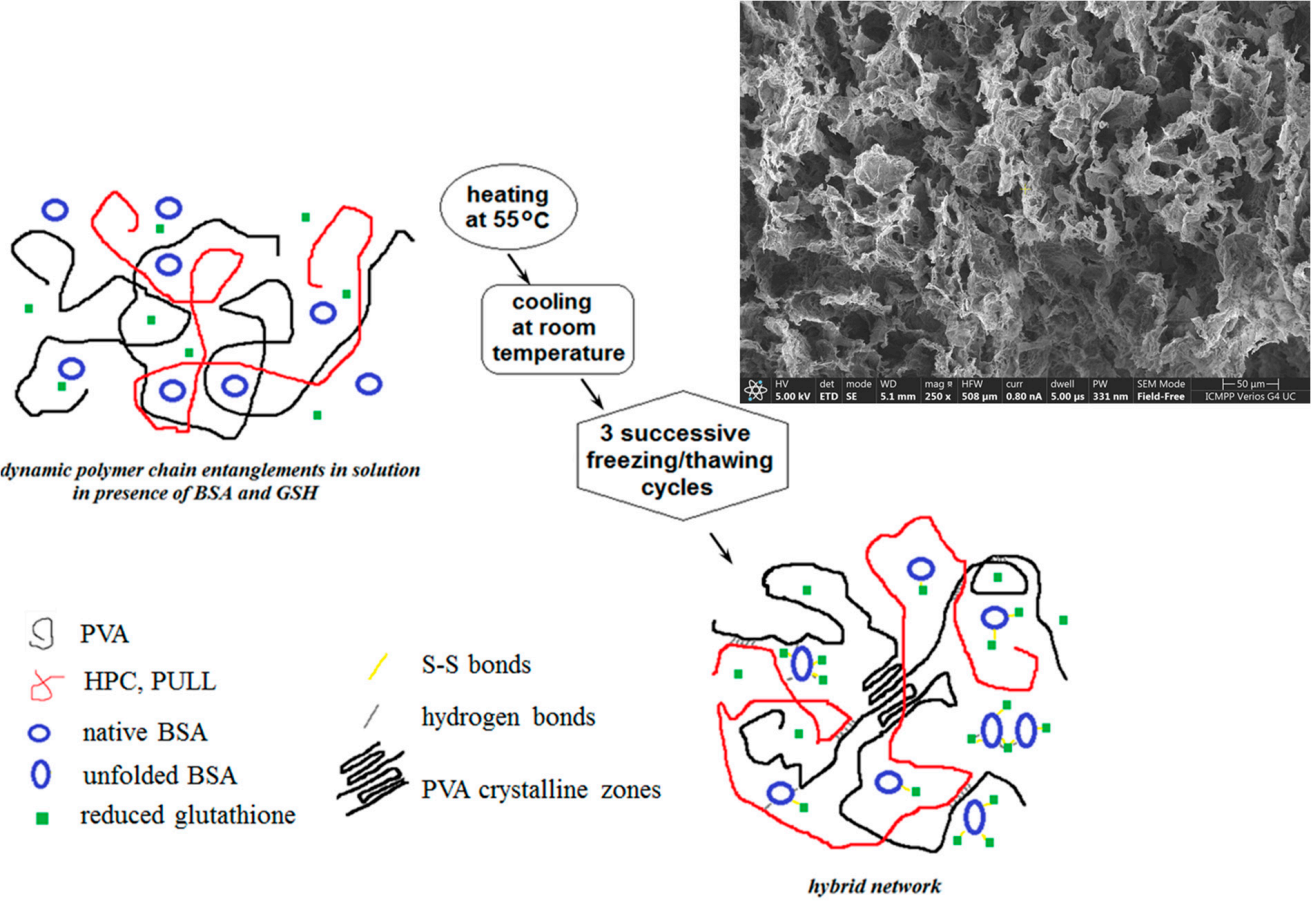
Schematic illustration of PVA-based hybrid hydrogel formation (adapted from [56,58]).

**Figure 4:**
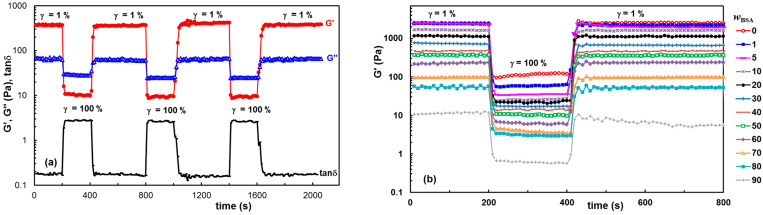
The self-healing behavior of PVA/HPC/BSA hydrogels illustrated through consecutive step strain measurements at 37 °C: (**a**) G′, G″ and tanδ for the hydrogel with 50% BSA in composition during successive runs of low (1%) and high (100%) strains; (**b**) G′ for different polymer/protein compositions (*w*_BSA_ is the weight percent of BSA in the polymer/BSA mixture) during the first run of strain (1%—↑ 100%—↓ 1%) [58].

In addition to experimental conditions, PVA characteristics (molecular weight, DH, tacticity, critical reduced concentration in aqueous solution) are taken into account for the design of hydrogels. This ecofriendly procedure was largely applied by different groups [3,4,7,15,18,29,30,33,55,56,58,61,69,70,71,72,73,74,75,76,77,78,79,80] to produce high-performance hydrogels, films, scaffolds, nanomaterials etc. By using natural/synthetic polymers and proteins (Figure 3), synergistic effects can be obtained [56,58,78]. Hydrogels with elastic and porous structures present self-healing ability (Figure 4) due to the occurrence of multiple interactions (as shown in Figure 3), with all components included in the network contributing to the overall behavior.

Physically crosslinked PVA hydrogels are often preferred materials for a variety of uses, especially in bio-related fields, because they exhibit a high degree of purity, and the design process is carried out under mild conditions. The use of the FT technique allows for the preparation of carriers used for drug incorporation and delivery, with a wide range of pore sizes and textures. Using solutions of PVA of different molecular weights and controlling the freezing/thawing parameters, carriers stable in aqueous solutions were obtained. The drug release is governed by swelling, diffusion or erosion mechanisms [66,78]. The release kinetics can be delayed by increasing the PVA density in the network [66], and the delivery of active compounds can be improved by synergistic combination with proteins [56].

**Figure 5:**
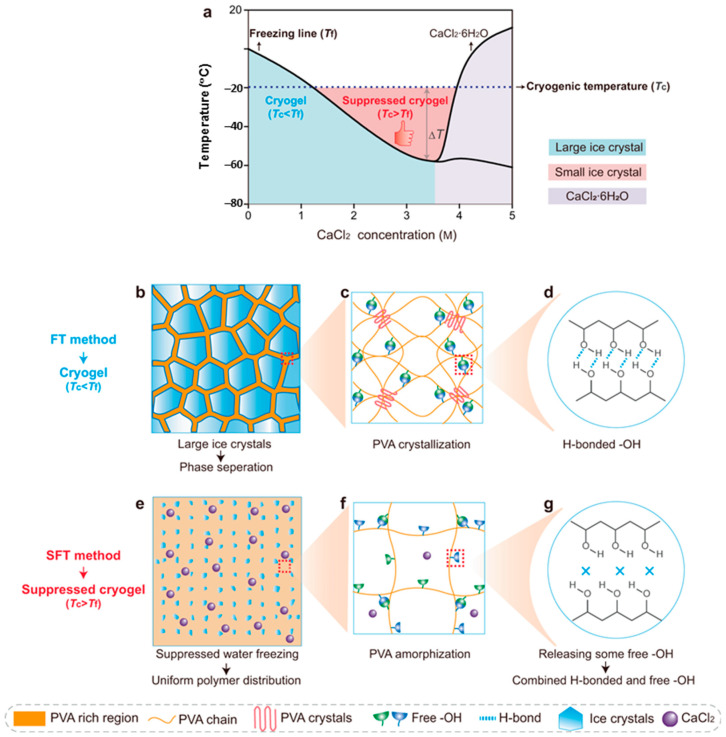
(**a**) A phase diagram of the PVA/CaCl_2_/H_2_O solution, and the design principle of conventional cryogels obtained by the FT method and suppressed cryogels formed by the SFT method based on the relationship between freezing temperature T_f_ and cryogenic temperature T_c_ (ΔT = T_c_ − T_f_). (**b**–**g**) The cryogenic state and the corresponding multiscale structures for conventional cryogels (**b**–**d**) and suppressed cryogels (**e**–**g**). Adapted with permission from [6], copyright 2023, Springer Nature.

Generally, cryogels adopt a semi-crystalline structure for on-demand functionalities. These structures formed into uncontrolled freezing fields present stiffness that limits their use as soft tissues, for which the modulus values are in the range of 1–100 kPa [82]. For robust tissues (tendons or cartilages), the modulus reaches higher values (>100 kPa), and such stiff structures can be obtained by the FT method. On the other hand, soft materials require low modulus values, between 1 Pa and 10 kPa [6]. To obtain ultrasoft hydrogels, the suppressed freezing/thawing method (SFT) was used, which involves the addition of anti-freezing salts to precursor solutions in order to lower the freezing point, T_f_, below the cryogenic temperature, T_c_ (Figure 5) [6]. 

**Figure 6:**
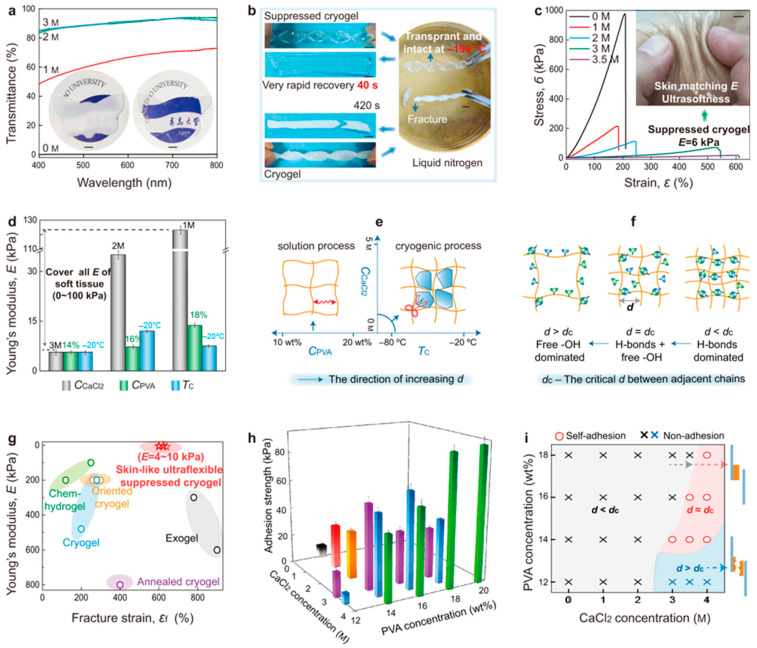
(**a**) The transmittance of cryogels and suppressed cryogels with a thickness of 2 mm (inset: photographs of opaque cryogels and transparent suppressed cryogels (3 M)). (**b**) The twisted cryogels and suppressed cryogels are placed into liquid nitrogen (−196 °C) and then converted back into ambient temperature (24 °C). (**c**) Stress–strain curves and the image of flexible suppressed cryogels (ultralow Young’s modulus E) closely contacted with skin. (**d**) Variations in Young’s modulus with CaCl_2_ concentration (CCaCl_2_) (C_PVA_ = 14 wt.%, T_c_ = −20 °C), PVA concentration (C_PVA_) (CCaCl_2_ = 3 M, T_c_ = −20 °C) and cryogenic temperature (T_c_) (C_PVA_ = 14 wt.%, CCaCl_2_ = 3 M). (**e**,**f**) A schematic presentation of the effect of C_PVA_, CCaCl_2_ and T_c_ on the microstructure of cryogels during the FT process (**e**), and the variation in free/H-bonding –OH groups with the distance (**d**) between adjacent chains (**f**). (**g**) A comparison of Young’s modulus versus fracture strain among different PVA hydrogels, including those of the chemically crosslinked hydrogel (chem-hydrogel), the cryogel treated using directional ice template or mechanical training (oriented cryogel), the cryogel treated using annealing (annealed cryogel), the hydrogel fabricated using solvent exchange (exogel) and the cryogel created using the FT and SFT strategies. (**h**,**i**) Variations in adhesion strength with C_PVA_ and CCaCl_2_ under T_c_ = −20 °C (the adhesion strength of <50 kPa was defined as non-adhesive nature). Error bars = standard deviation (*n* = 6) in (**d**,**h**). Scale bars: 5 mm in (**a**–**c**). Adapted with permission from [6], copyright 2023, Springer Nature.

The presence of salts suppresses the ice growth during freezing. After the thawing step, the amorphous structure prevails, characterized by the coexistence of free and hydrogen bonding –OH groups, which triggers characteristics similar to skin/tissues (Figure 6): high softness (Young’s modulus < 10 kPa), stretchability (~600%) and transparency (~92%), self-adhesion and fast self-healing (<0.3 s), high ionic conductivity (2.94 S m^−1^ at 20 °C), anti-freezing (−58 °C) and water retention due to the ionic hydration (water loss of ~10 wt.%, much lower than of conventional cryogels, i.e., ~74 wt.%) [6].

The suppressed networks obtained using the SFT method present high transparency (Figure 6a) with a transmittance of 91.6% at a wavelength of 600 nm, while those obtained by the FT procedure are more opaque. At very low temperature (−196 °C, liquid nitrogen), the SFT structures maintained their form and transparency, whereas the FT hydrogels easily fractured and became opaque. During thawing at 24 °C, the SFT gels recovered their flexibility in 40 s, while the FT networks needed 420 s (Figure 6b). The main characteristics of SFT were well evidenced by Zhang et al. [6] (Figure 6).

### 3.2. Non-Cryogenic Physical Gelation

PVA chains dissolved in aqueous solutions or mixtures of water and organic solvents spontaneously associate to form a 3D network. From thermodynamic considerations, PVA solutions simultaneously undergo spinodal decomposition and gelation [35,64,83]. Based on this behavior, PVA networks were created by dehydration after casting the PVA solution, without adding any crosslinker. Thus, the PVA aqueous solution was poured into a mold and stored at room temperature for a few days [37]. The polymer chains entangled faster as the water molecules were removed, favoring intra- and intermolecular hydrogen bonds, and thus, a hydrogel structure was created. Other miscible solvents, such as alcohols, may be added to speed up the water removal process [84]. The degree of crystallinity was increased by incubation into aliphatic alcohols (which are non-solvents for polymers), when PVA/water H-bonds were replaced by PVA/PVA intermolecular H-bonds. By using this procedure, electrospun fibers were stabilized against their dissolution in water [85]. Efficient dehydration was also obtained by adding various drying agents, such as acetonitrile, acetone ethylene glycol or glycerol [84].

In a NaOH solution (about 4% *w*/*w*), PVA is able to undergo in situ crystallization [86]. The HO^−^ ions from NaOH deprotonate –OH groups from the polymer chains, and O^−^ groups interact with free Na^+^ from the solution and form a complex, favoring the stretching and alignment of PVA chains that organize into crystalline domains. Also, the ester groups from acetate moieties hydrolyze in the presence of HO^−^. In moderate alkaline solutions, the two-phase aqueous PVA system generates a physical network by 3D printing in embedding media through layer-by-layer adhesion. The salting-out effect induced by Na_2_SO_4_ addition improves the hydrogen bonds and ensures the shape fidelity of the printed structures [35].

A robust and versatile route for PVA fibers from a nanometer to micrometer scale is the electrospinning technique using entangled solutions [36,48,87,88]. The main parameters for optimizing the nanofiber characteristics are the initial polymer concentration, PVA tacticity, applied voltage and tip-to-collector distance [89]. Increasing the applied electrical field or the addition of AlCl_3_ decreased the diameter of nanofibers [90]. Electrospun fibers present high potential for applications in the biomedical field, such as personalized wound dressings, smart coatings with high specific contact surface, biosensors, tissue engineering, drug delivery or cancer therapy, filtration materials etc.

### 3.3. Chemical Crosslinking of PVA

Chemical crosslinking, involving the creation of chemical bonds between different PVA macromolecules, is frequently used to improve PVA properties, making it more valuable material for various applications: pervaporation membranes, food packaging, reverse osmosis, desalination, wound dressing, drug delivery or fuel cells [91]. PVA crosslinking reduces the hydrophilic characteristics of polymers due to the diminution of the number of hydroxyl groups. Using specific crosslinking agents, a network structure is formed, which is not further soluble in water or other solvents but swells and absorbs a high amount of water of other small molecules. The chemical crosslinking occurs through –OH groups of PVA either by classical reactions (esterification, etherification, carbamation, initiation of radical polymerization) or modern methods (click chemistry, bioconjugation, creation of dynamic bonds) [13,92,93]. PVA crosslinking methods are well presented in comprehensive reviews (for example, [91,94,95,96]). The chemical networks present higher glass transition temperature and physicochemical stability and improved viscoelastic and mechanical characteristics as compared with physical PVA hydrogels [97,98,99].

Chemical bonds can be realized through multifunctional molecules, such as aldehydes (formaldehyde [100,101,102], glyoxal [103,104] or glutaraldehyde, GA [27,105,106,107]), urea oligomers [108], dicarboxylic acids [91,109], epichlorohydrin [27], inorganic compounds (such as borate-containing species [110,111,112,113]) or gamma irradiation [114,115].

In acidic solutions at room temperature, the –OH groups of PVA react with the aldehyde group (–CHO) of GA, resulting in gel structures with acetal or hemiacetal bridges as crosslinking points. By adjusting the concentration and molecular weight of PVA or the GA/PVA ratio, the hydrogel characteristics can be modulated in a reproducible way [105,116,117,118,119,120]. Membranes with a gradient acetalized PVA structure (decreasing the degree of crosslinking from the surface to inside) were prepared by casting the PVA aqueous solution followed by surface crosslinking with GA [121,122]. The permeability and selectivity of membranes can be tuned by the reaction time and GA concentration. These transparent membranes have shown excellent mechanical performances and good water resistance, being suitable for cell encapsulation or bioseparation.

The chemical modification or functionalization of PVA through OH groups (etherification, esterification, acetalization, imidization, amination, phosphorylation, sulfonation, azidation) can lead to the production of membranes with improved efficiency [123]. Also, the –OH groups act as active sites to initiate graft polymerization [123,124].

Transparent and thermoreversible physical gels were obtained by adding monocarboxylic acids (having different lengths of an alkyl chain, i.e., formic, acetic, propionic or butyric acid) during the dissolution of PVA in dimethyl sulfoxide (DMSO) at high temperature (95 °C), followed by aging in rest conditions, at room temperature [125]. DMSO is a polar aprotic solvent which forms hydrogen bonds with –OH groups of polymers, avoiding the occurrence of PVA/PVA interactions. In the presence of monocarboxylic acid, the oxygen atom of DMSO preferentially establishes hydrogen bonds with carboxyl groups of the acid, whereas the –OH groups of PVA become available to generate intra- and intermolecular polymer/polymer interactions. Also, monocarboxylic acids are involved in hydrogen bonds with PVA, contributing to the physical network formation. The gelation and mechanical properties were influenced by the concentration and alkyl chain length of the acid and aging time. Thus, high strength (2.2 MPa), tensile elongation (720% at break) and toughness (7.7 MJ/m^−3^) values were reported for these hydrogels. The dissolving temperature range was broadened by adding formaldehyde, which increases the hydrophobicity and crystallinity of acetalized fibers [126].

PVA crosslinked with polycarboxylic acids is a promising non-toxic and available chemical route. PVA hydroxyl groups undergo esterification with dicarboxylic acids following nucleophilic addition reaction and forming 3D networks with high stability due to the multiple ester linkages. The hydrophilic groups present in the network interact with water molecules, whereas hydrophobic groups improve the interaction with drugs or other hydrophobic molecules; thus, membranes for separation processes can be designed [91]. The main factors influencing the hydrogel properties are as follows: crosslinker nature and concentration, the duration of reaction and the temperature of curing. The induced properties refer to the increase in thermal stability, enhancement in mechanical properties, decrease in hydrophilicity and improvement in biological properties [91,109]. Among the most used polycarboxylic acids, the following compounds can be mentioned: citric acid [127,128,129], tartaric acid [130], L-malic acid [131], sebacic acid [132] etc.

In aqueous solutions, near the overlap concentration, a PVA borate complex is formed, and its negative charges are screened by free Na^+^ cations [132]. With increasing the polymer or borax concentration, a crosslinking reaction occurs through a so-called “di-diol” complexation between one borate ion and two diol units of PVA. The reversible sol–gel transition is sensitive to balance between the PVA crosslinking induced by the borate ions and the electrostatic repulsions by the charged complexes along the macromolecular chains. In the sol state, two relaxation modes were evidenced by dynamic light scattering (DLS): a fast mode corresponding to the self-diffusion of polymer chains and a slow mode corresponding to the relaxation of PVA clusters. It was suggested that the low mode is due to the hindered motions of associative chains, where there are either weak segment–segment interactions in a poor solvent or strong electrostatic interactions between the charged entities. In the gel state, only a fast relaxation mode was observed by DLS, corresponding to a collective motion of the transient network. In another approach, a series of derivatives were synthetized by incorporating thiol, cysteine 1,2-aminothiol or aminooxy side chains into PVA, able to further act as crosslinking agents for hyaluronic acid [133]. In a recent paper [134], new applications were tested for PVA networks obtained in the presence of boric acid, i.e., the design of flexible and radiation shielding devices with potential interest in the fabrication of protective equipment for astronauts.

Quercetin is also a promising crosslinking agent for PVA. Using a 50/1 (*w*/*w*) PVA/quercetin ratio, the mechanical strength, water resistance and antioxidant ability of the PVA hydrogel was significantly improved [135].

Chemical crosslinking induces some adverse effects such as toxicity or undesirable secondary reactions [98,116]. Despite these inconveniences, the hydrogels formed by chemical bonds may be a good choice for articular cartilage repair [136]. Also, materials rich in H-bonds can provide a shielding against heavy ion particles. 

### 3.4. PVA Hydrogels Obtained by Combined Methods

**Figure 7:**
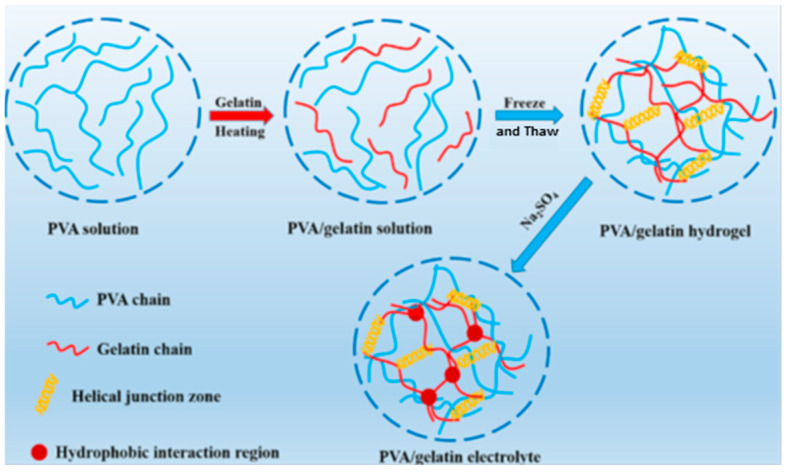
Schematic illustration of PVA/gelatin gel electrolyte DN formation, involving first network of PVA via FT cycles and second network of gelatin via Hofmeister effect. Adapted with permission from [137], copyright 2023, Elsevier Ltd.

**Figure 8:**
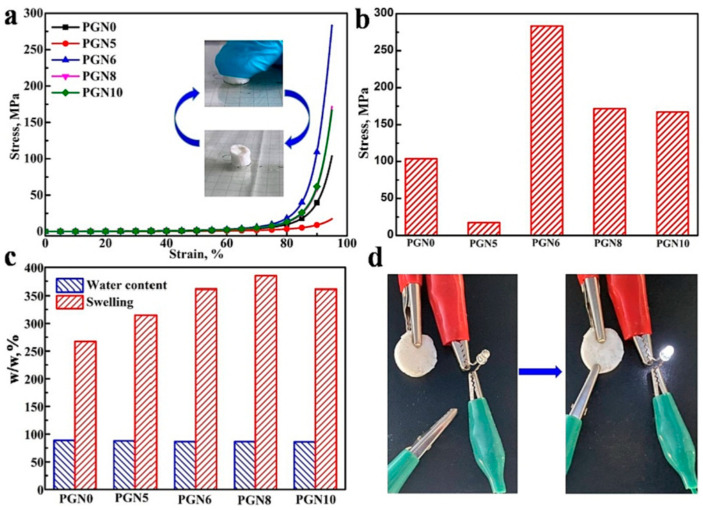
(**a**) Compressive curves of DN hydrogel with various gelatin contents; (**b**) effect of gelatin content on strength; (**c**) water content and swelling ratio of DN with various gelatin contents; (**d**) hydrogels were connected into circuits with a light-emitting diode (LED). Adapted with permission from [137], copyright 2023, Elsevier Ltd.

**Figure 9:**
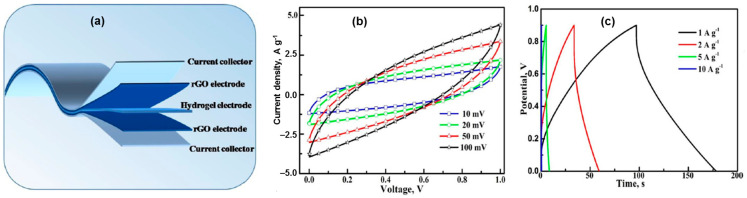
The electrochemical performance of the PVA/gelatin DN: (**a**) a schematic diagram of the assembled supercapacitor; (**b**) CV curves at variable scan rates from 10 to 100 mV/s and (**c**) galvanostatic charge/discharge profiles of the device at various current densities from 1 to 10 A/g. Adapted with permission from [137], copyright 2023, Elsevier Ltd.

Versatile PVA/gelatin porous 3D double networks (DNs) with excellent mechanical performances and electrochemical properties were obtained by combined methods [137]. The first PVA network was formed by applying the FT cycles, and the second network of gelatin was generated via the Hofmeister effect (Figure 7). The resulting electrolyte gels presented good mechanical properties, being able to support high deformations: for a ratio of gelatin/PVA = 1/6, a tensile strength value of 1.0 MPa, an elongation at break of 582% and fracture energy of 3.2 MJ/m^3^; also, a high value of compressive strength was registered (283 MPa for 95% strain). The water absorption ability was improved by increasing the gelatin content, and Na_2_SO_4_ addition into DNs induced high conductivity (Figure 8). The assembled supercapacitor of reduced graphene oxide (rGO) gel as electrodes and DNs as electrolyte gel showed good electrochemical performances; thus, a value of 83.6 F/g was obtained for specific capacitance at a current density of 1 A/g (Figure 9).

**Figure 10:**
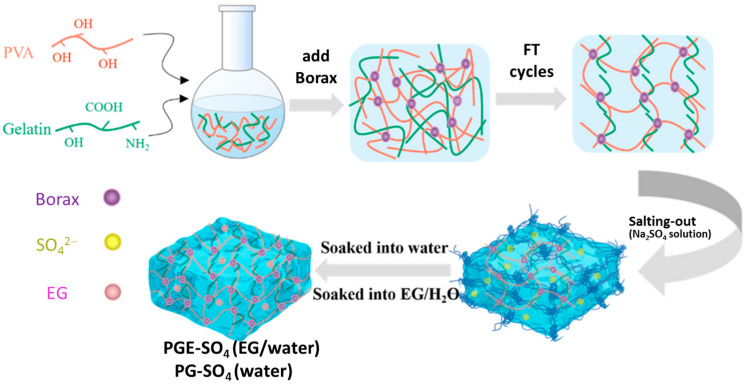
Schematic presentation of PVA/gelatin hydrogels. Adapted with permission from [2], copyright 2024, Elsevier B.V.

**Figure 11:**
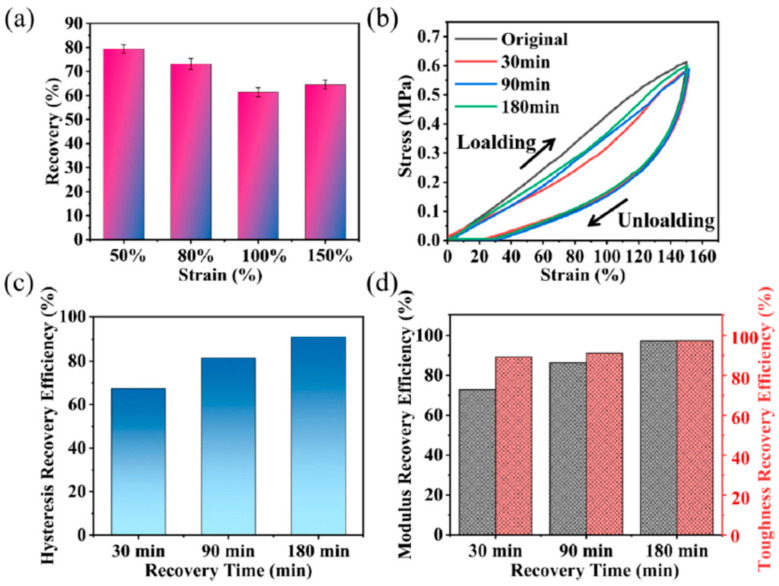
Recovery rates at different strain tensile cycles of PGE-SO_4–1.5_ hydrogel without interval time (**a**) and tensile stress–strain curves at different intervals (**b**). Recovery efficiency at different intervals for hysteresis energy (**c**), elastic modulus (gray) and toughness (red) (**d**). Adapted with permission from [2], copyright 2024, Elsevier B.V.

**Figure 12:**
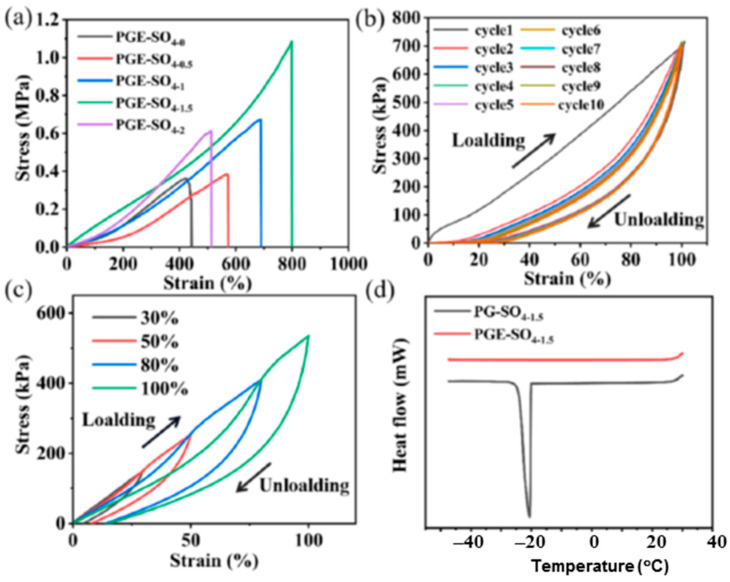
Mechanical behavior of hydrogels at −20 °C. (**a**) Tensile stress–strain curves of PGE-SO_4–X_ hydrogels prepared by FT and immersed for 24 h into Na_2_SO_4_ solutions of X mol/L concentration (X = 0.5, 1, 1.5 and 2 mol/L). Ten stretch loading–unloading cycles under 100% strain (**b**) and tensile loading–unloading curves at different stresses (**c**) of PGE-SO_4–1.5_. (**d**) DSC curves of PGE-SO_4–1.5_ and PG-SO_4–1.5_ hydrogels. Adapted with permission from [2], copyright 2024, Elsevier B.V.

A similar strategy of combined FT and the salting-out effect was used to prepare PVA/gelatin hydrogels in the presence of borax and Na_2_SO_4_ water (denoted PG-SO_4_, Figure 10) and ethylene glycol (EG)/water mixtures (denoted PGE-SO_4_, Figure 10) for their application as flexible electronic devices [2]. The DN presented good mechanical properties (such as fracture tensile strain of 1281%, tensile strength of 1.12 MPa and toughness of 7.19 MJ/m^3^) and high recovery rates of different parameters (97.36% for the Young modulus and 97.47% for toughness, reached after 180 min), as shown in Figure 11 and Figure 12 [2].

Strong, tough and elastic PVA/polyacrylamide (PAm) DNs able to mimic the characteristics of the native articular cartilage were prepared by in situ polymerization followed by a treatment with various anions (Cit^3−^, SO_4_^2−^, and Cl^−^) [138]. The best performances were observed in the presence of Cit^3−^: tensile strength of 18.9 MPa, compressive strength of 102.3 MPa, tensile modulus of 10.6 MPa, compressive modulus of 8.9 MPa and roughness of 66.2 MJ/m^−3^, with good bioadhesivity, with the hydrogel being able to promote chondrocyte proliferation.

The water solubility of PVA nanofibers limits their application. Physical or chemical crosslinking is used to increase stability in an aqueous environment. For use as filter membranes or adsorbing materials, crosslinked PVA nanofiber mats with porous shape hence provide significant relevance. It was demonstrated that PVA fibers are not crosslinked by formic acid or glutaraldehyde vapors. PVA-based fibers can only be crosslinked by GA in solution; nevertheless, they are unstable in water. It was demonstrated that a thermal treatment is appropriate; PVA fibers acquire water resistance [139]. Also, PVA nanofibers crosslinked with GA in organic solvents were able to preserve the morphology and mechanical properties in water or after soaking into extreme conditions, for example, in hot water or a strong acid/alkaline environment at high temperature for a long period of time [140].

The resistance to the hydrolysis of electrospun PVA/chitosan membranes was improved by using GA as a crosslinking agent in the presence of hydrochloric acid. The membrane presented good filtration efficiency and antibacterial properties, being suitable as a protective mask [141]. Also, a significant increase in filtration efficiency was obtained by crosslinking PVA nanofibers with maleic acid [142]. PVA partially crosslinked with GA was transferred into a reactor (a long hot tube) and then electrospun before gelation. Thus, the mechanical properties and thermal stability were improved as compared with simple PVA fibers or those crosslinked GA vapors [143].

A synergistic effect was observed using two crosslinking agents, glutaraldehyde (GA) and ammonium persulfate (APS); water resistance and thermal stability were considerably improved [144]. Another strategy was to investigate the synergy in the thermal and mechanical properties of PVA by using multiple methods, such as chemical crosslinking (using glutaric acid) and dual reinforcing agents (tungsten disulfide nanotubes and carboxylated multiwall carbon nanotubes) [145] or crosslinking with tartaric acid followed by microwave irradiation or conventional heating methods [146]. The use of some chemical crosslinkers (such as GA, epichlorohydrin) generates toxic microenvironments and decreases hemocompatibility. The material may stimulate thrombus formation, which is influenced by the hydrogel surface properties [147,148].

The main techniques used for the characterization of PVA hydrogels are presented in Table 1.

Using PVA hydrogels, the drug release profile was frequently altered by an initial burst effect or a lag phase; these effects can be avoided by the introduction of surface crosslinked layers into PVA networks. By a careful selection of surface crosslinking parameters (crosslinker concentration, exposure time), the optimum thickness and crosslinking density of the surface layers can be obtained. Thus, the initial burst release effect could be removed in order to achieve reproducible delayed release [149].

The complex structures obtained during crosslinking determine a variety of viscoelastic responses at large-amplitude oscillatory shear (LAOS). PVA/borax physical gels with temporary junction points displayed Giesekus-like linear response, shear thinning at large strains and a gradual strain stiffening due to the stretching of segments between two crosslinks. Chemically permanent crosslinks of PVA and hyaluronic acid (HA) flow apart, along with the stretching of crosslinked and uncrosslinked chains, exhibit intra-cycle strain stiffening and shear thickening at large strains and present a weak frequency variation in the viscoelastic moduli in the linear range [150].

## 4. Applications of PVA-Based Hydrogels

The high scientific and applicative interest for PVA is due to its structural versatility and tunability to produce materials in various forms, hydrogels, membranes, nanofibers, films etc., in most cases, in combination with other micro- and macromolecular compounds. There are many published reviews that emphasize the special performances of this polymer. In the next section, some recent achievements will be pointed out.

### 4.1. Wound Dressings

Over the last few decades, efforts have been oriented to create new porous polymeric membranes that meet the requirements needed for skin wound healing. Wound exudates can be absorbed and retained by porous hydrogels, which promote fibroblast growth and keratinocyte migration. Complete epithelialization and wound healing depend on these processes [97,136,151,152]. Furthermore, the network structure with tight mesh size ensures the protection against infection acting as a barrier that avoids the invasion of microorganisms and bacteria; it keeps the raw wound clean, reducing the pain until complete healing. The skin is the largest organ of the body that ensures tactile sensation, thermoregulation and also the production of vitamins and metabolites. Hydrogels have the flexibility to adapt to any shape of wounds, allowing for the entrapment of bioactive compounds into the pores and their transport to the wound surface. It was shown that PVA-based networks are suitable materials for wound dressing applications [33,152,153]. Generally, hydrogels with a single component did not fulfill all characteristics, for example, poor biocompatibility or low mechanical strength. A lot of research is carried out on composite or hybrid hydrogel membranes [33,56,59,78].

PVA/cellulose hydrogels are characterized by strong H-bonds between the two polymers that improve the mechanical properties and thermal stability [154,155,156,157]. In addition, they present hydrophilicity and biocompatibility [158]. Using molecular dynamic simulations, the interactions between PVA and cellulose were investigated. The high binding energy and cohesive energy density explain the superior mechanical properties of the PVA/cellulose composite hydrogels [159].

However, the lack of antibacterial activity limits the use of PVA-based hydrogels in wound dressing applications. Antibacterial properties can be achieved by using antimicrobial agents, such as drugs [4,33,56], peptides [160,161], essential oils [36,162,163] or metal/metal oxide nanoparticles [60,164]. Green synthetized silver nanoparticles incorporated into PVA materials improve the antimicrobial activity [164,165,166,167,168]. The antimicrobial activity can also be induced through plasma-activated hydrogel therapy, with high efficiency against *Escherichia coli* and *Pseudomonas aeruginosa* and with lower efficiency against *Staphylococcus aureus* [169].

PVA/polydopamine (PDA)/TA composite hydrogels with adhesive, antibacterial and antioxidant properties were prepared by the one-pot method. The maximum skin adhesive strength value was 75.7 kPa, and the antibacterial efficiency was up to 99% against two pathogenic microbes: *Escherichia coli* and *Staphylococcus aureus* [170]. Deformable or laser-engravable electroluminescent devices were designed by including biosensors based on PVA/PDA/graphene oxide hydrogels with optical, photothermal and mechanical tenability, able to monitor human motion (linear sliding or bending) [171].

Biocompatible PAm/PVA-based hydrogels in combination with antimicrobial poly(ionic liquid) (poly(1-glycidyl-3-butylimidazolium salicylate)) and PDA-coated nanosheets were prepared for wound dressing application. The composite hydrogels presented antimicrobial activity (over 95% against *Escherichia coli*, *Staphylococcus aureus* and *Candida albicans*), antioxidant and anti-inflammatory properties and accelerated wound healing ability (wound closure in 14 days) [60].

PVA/chitosan multifunctional hydrogels reinforced by nanocellulose and CuO-Ag nanoparticles provided mechanical stability to the wound site and presented a suitable antibacterial environment for wound healing [172]. Another recent study described biocompatible PVA/chitosan composite hydrogels with a bidirectional porous structure by using polyethylene glycol (PEG) as the pore-forming agent and rGO-PDA@ZIF-8 as antibacterial nanofiller. In vivo wound treatment has shown that wound healing is promoted in the presence of this composite hydrogel [173].

Biocompatible electrospun nanofibers composed of PVA, chitosan and usnic acid with average sizes between 30 and 40 nm and antimicrobial activity against *Staphylococcus aureus* were reported by Stoica et al. [174].

A photocrosslinkable wound dressing was created using PVA/carboxymethyl chitosan foam as layer support and gelatin methacrylate (GelMA) mixed with tannic acid (TA) as a second layer [175]. The hydrogel composed of these two layers with different structures and functionalities demonstrated broad antibacterial activity against Gram-positive and Gram-negative bacterial strains. TA contains polyphenol groups that lead to compact crosslinking and induces bioadhesivity, antioxidant, anti-mutagenic, anti-carcinogenic and antibacterial properties [176].

Soft and flexible wound dressings were prepared by the FT method using solutions of PVA, human-like collagen and carboxymethyl chitosan mixtures in the presence of a pore-forming agent (Tween80) [177]. The hydrogels presented good biocompatibility, degradability and hydrophilicity and were recommended as hemostatic materials for skin wound healing.

Composite nanofiber membranes with average fiber diameters between 238 nm and 595 nm were fabricated using 12.5% gelatin and 15% PVA (80/20; 85/15 and the optimum ratio of 10/90), 3% bioglass, and for crosslinkers, either 15% citric acid or 15% citric acid was used in the presence of 10% urea. The tensile strength values were determined to be between 10.28 MPa (for 80% PVA) and 8.46 MPa (for 90% PVA). The membranes presented good biocompatibility and anti-inflammatory effects, with high potential for biomedical applications as wound dressings [178].

A therapeutic electroconductive wound dressing was designed by integrating β-cyclodextrin-embedded silver nanoparticles with antibacterial activity in a porous PVA matrix loaded with free β-cyclodextrin for enhancing the mechanical strength and β-glucan grafted with hyaluronic acid which improves biocompatibility. The multifunctional composite hydrogel presented hemostatic properties and promoted the in vitro proliferation of fibroblasts, accelerating the wound healing [179]. Another tested strategy to promote wound healing was the addition of therapeutic herbal ingredients to PVA hydrogels obtained by alkali crosslinking. Also, by applying this method, the cell viability, angiogenesis and collagen deposition were enhanced, and the inflammatory response was diminished [180].

### 4.2. High-Performance PVA-Based Hydrogels for Tissue Engineering

**Figure 13:**
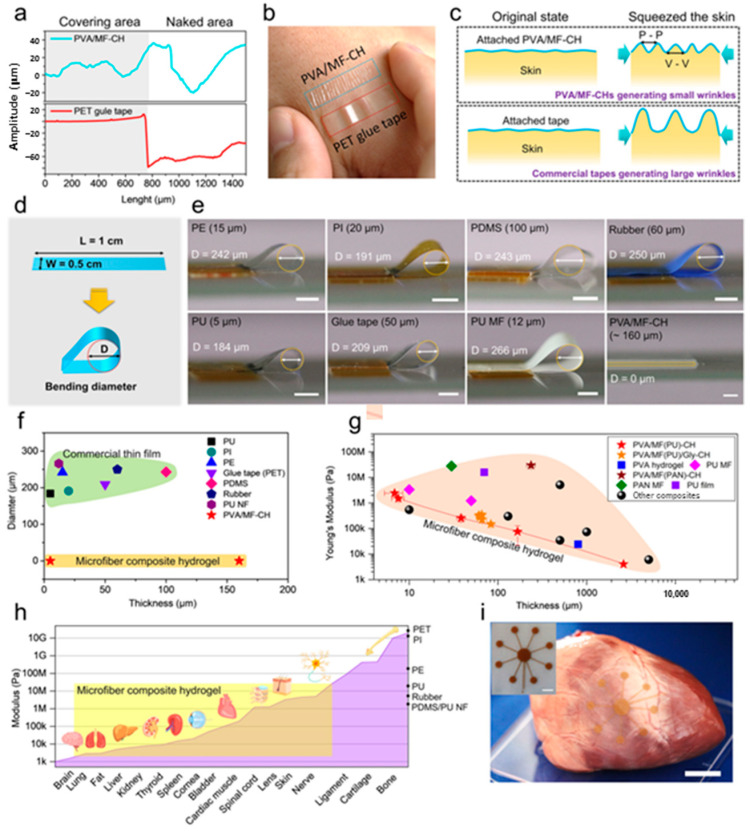
The tunable conformability and flexibility of PVA microfiber composite hydrogels (PVA/MF-CH): (**a**) The surface roughness of the artificial skins of PVA/MF-CH and polyethylene terephthalate (PET) glue tape. (**b**) A digital image displaying wrinkles generated from the PVA/MF-CH and PET glue tape induced by squeezing the skin. (**c**) A schematic wrinkle-generating mechanism of the skin covered by PVA/MF-CH and PET glue tape when squeezing. (**d**) A schematic diagram of the softness evaluation with a bending diameter (D). (**e**) Digital images of bending diameters generated from different materials. Specimen size: 1 cm (L) × 0.5 cm (W). (**f**) The diameter of the bending circle generated in different materials. P-P and V-V mean the distance between two peaks and two valleys, respectively. (**g**) The Young’s modulus and thickness of different materials. (**h**) The modulus matching a range of PVA/MF-CH with biological tissues and organs. (**i**) A digital image of a porcine heart with an attached PVA/MF-CH-based bioelectrode. The inserted picture is the PVA/MF-CH-based bioelectrode. PAN = polyacrylonitrile; PE = polyethylene; PU = polyurethane; PDMS = polydimethylsiloxane; PI = polyimide. Adapted with permission from [5], copyright 2023, Springer Nature.

**Figure 14:**
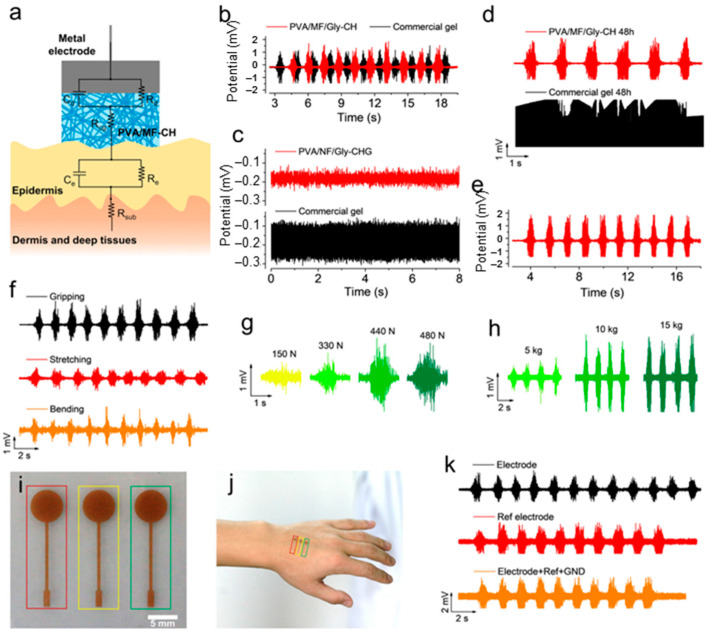
EMG biosignal monitoring: (**a**) A schematic of the equivalent circuit model used for monitoring the EMG biosignals. At the electrode level (top three elements), Rd is the charge transfer resistance, C_d_ is the double-layer capacitance and R_cg_ is the resistance of the composite gel. At the skin level (bottom three elements), R_e_ and C_e_ are the epidermal resistance and capacitance, respectively, and R_sub_ is the resistance of the dermis and deep tissues. (**b**) A performance comparison of EMG biosignals collected by the electrode composed of PVA/MF/Gly-CH and a commercial gel. (**c**) The background noises of the electrode composed of PVA/MF/Gly-CH and a commercial gel. (**d**) A performance comparison of the electrode composed of PVA/MF/Gly-CH and a commercial gel for the monitoring of EMG biosignals after 48 h. (**e**) The performance of the electrode composed of PVA/MF/Gly-CH for the monitoring of EMG biosignals after 7 d. (**f**) EMG biosignals of the forearm are generated from different gestures. (**g**) EMG biosignals of the forearm are generated from different gripping forces. (**h**) EMG biosignals of the bicipital muscle of the arm lifting the different masses of the object. (**i**) A tri-electrode system comprising PVA/MF/Gly-CH for the monitoring of EMG biosignals. (Electrode in red rectangle, GND in yellow rectangle and R_ef_ electrode in green rectangle). (**j**) A digital image of the hand with an attached tri-electrode system comprising PVA/MF/Gly-CH. (**k**) The EMG biosignals of the forearm collected by the PVA/MF/Gly-CH-based bioelectrode. MF = microfiber; CH = composite hydrogel; Gly = glycerol; EMG = electromyography. Adapted with permission from [5], copyright 2023, Springer Nature.

The fabrication of substitute materials for articular cartilage using low-frictional hydrogels revealed a high applicative potential. PVA-based hydrogels are suitable materials for fabricating flexible bioelectronics with the required softness, stretchability, fracture toughness, biocompatibility with tissues and ionic conductivity, as an appropriate interface to bridge thin-film electronics with soft tissues. Architectures inspired by the extracellular matrix (ECM) can serve as scaffolds for tissues and organs. During their preparation, the mechanical properties of hydrogels can be easily customized for maintaining the structural and functional integrity of damaged tissues and organs. Ultrathin (<5 μm) and ultrasoft composite microfibers (MFs) with tunable modulus values ranging from ~5 kPa to tens of MPa (as found for the most biological tissues and organs) were prepared by embedding electrospun microfibers into a hydrogel structure (Figure 13) [5]. Glycerol and salt ions were incorporated in the composite network, determining high ionic conductivity and anti-dehydration tendency. Due to its high dielectric constant (about 42.5), the role of glycerol is to weaken the columbic attraction between the polymer chains and the cations or anions of the salts [181]. PVA microfiber composite hydrogels (PVA/MF-CH) are promising for constructing flexible electronics to monitor biosignals (Figure 14) [5].

Tough and porous physical hydrogels with good mechanical and anti-swelling properties were prepared by using PVA and sodium phytate (PANa). The addition of PANa into the PVA matrix enhances the intermolecular H-bonds and favors the formation of a conductive network sensitive to strain, suitable for implantable biomaterials [182].

Bioinspired muscle-like conductive hydrogels were prepared using the PVA matrix reinforced with cellulose nanofibrils (CNFs), adding Ag^+^ as the conducting medium and hydrolyzed lignin for interfacial binding [183]. These double physical composite networks presented high toughness, anisotropic strength and conductivity. Another study was carried out on PVA films reinforced with CNF and TA [184]. The hydrogen and ester bonds established in this ternary mixture considerably improved mechanical and thermal characteristics as compared with the neat PVA film.

PVA-based nanofibrous bilayer scaffolds were designed with tetraethyl orthosilicat (TEOS) used as a crosslinker by combining a bottom layer of a 3D porous interconnected matrix of PVA/gelatin with a top layer by electrospun PVA/bacterial cellulose [185]. These scaffolds presented a high surface area that facilitated cell attachment and supported tissue growth and integration, also showing good antibacterial activity.

A dual dynamic covalent crosslinked hydrogel was prepared from PVA and 3-aminophenylboronic acid modified hyaluronic acid. This functional hydrogel provided injectability, self-healing behavior and anti-inflammatory effects, being of interest for treating osteoarthritis [186]. Another strategy was the chemical modification of PVA and silk fibroin (SF) by grafting with glycidyl methacrylate (GMA), in order to produce injectable biphasic hydrogels, PVA-*g*-GMA/SF-*g*-GMA [187]. These hydrogels provided porous structure with good mechanical properties, biodegradability and biocompatibility, being suitable for meniscus scaffolds.

### 4.3. Sensors

The design of highly sensitive hydrogels for sensing applications represents a challenge for researchers [62]. The strain sensor sensitivity is usually lower for hydrogels as compared with traditional strain sensors based on elastomers, and it is influenced by the correlation between the changes in electrical conductivity and the network deformation. Thus, a great challenge for various groups has been to design systems with high sensitivity. In this regard, PVA-based hydrogels with tunable mechanical properties may be appropriate for smart and flexible electronic devices for implantable sensors [188,189,190,191,192]. Shape memory materials attracted high interest for academic or applicative purposes. They present the ability to change their shape from one or more temporary supramolecular structure to an equilibrium shape, in response to external stimuli. This feature is due to the dynamic and reversible intermolecular interactions of such complex structures under the action of temperature, light, pH, ionic strength, redox agent, specific (bio)molecules etc. [193].

Furthermore, conductive hydrogels are promising candidates for flexible electronic devices. The inability of conventional conductive hydrogels to incorporate into a single system with important characteristics, such as biocompatibility, underwater sensing and high sensitivity under low deformation, significantly limits their use in aqueous environments [194]. A synergistic combination of PVA and tannic acid (TA) self-assembling properties leads to shape memory PVA-based hydrogels with amorphous structure and unique properties [38]. These networks with tunable hydrogen bonds (H-bonds) were prepared by physical crosslinking. Multiple H-bonds between PVA and TA mixtures lead to gelation at room temperature and their coagulation at high temperatures. The strong H-bonds between PVA and TA act as “permanent” crosslinks, and the weak but many H-bonds between PVA chains function as “temporary” crosslinks [195,196]. These PVA/TA hydrogels present good mechanical properties, such as excellent mechanical strength (up to 16 MPa [38], high elongation (up to 1100% [195]) and stretchability (≈1000% [38]) values. In addition, the physical interactions between PVA and TA allow for reversible breakage and the reformation of the network, exhibiting shape memory [38,194,195], self-healing ability [194,197], good stretching and anti-swelling characteristics, long-term stability in an aqueous environment and ability to detect human motions [194,197]. The PVA/TA supramolecular networks demonstrate electrical and mechanical performances, with a conductivity (κ) of 5.5 × 10^−4^ S/cm and gauge factor (GF) of 1.3 in dry and κ = 5 × 10^−4^ S/cm and GF = 1.2 in wet conditions, respectively [124], as well as a maximum tensile strength value of 104.2 MPa, Young’s modulus of 3.53 GPa and toughness of 395.2 MJ/m^3^, slightly higher than those of spider silk (354 MJ/m^3^) [198]. Another study reported on PVA/sodium glycinate hydrogels with high strain sensitivity (GF = 1.76) and electrical conductivity (4.85 S/m), as well as cytocompatibility and swelling resistance, obtained by the FT method [199]. These networks are suitable for the manufacture of wearable sensors.

PVA and phytic acid (PA) in the presence of glycerol form physical networks with anti-freezing properties and improved fatigue resistance and toughness. This hydrogel was tested as a sensor for monitoring human joint motion [200]. DN structures of PVA/PA (which generate the first network) and ethyl acrylate/3-(methylacrylamide) propyl trimethylammonium chloride (forming the second network) were created, presenting high antibacterial activity and conductivity (up to 9.1 S/m), adhesive strength up to 71 kPa and strain sensibility, as flexible sensors in detecting human motion [201].

**Figure 15:**
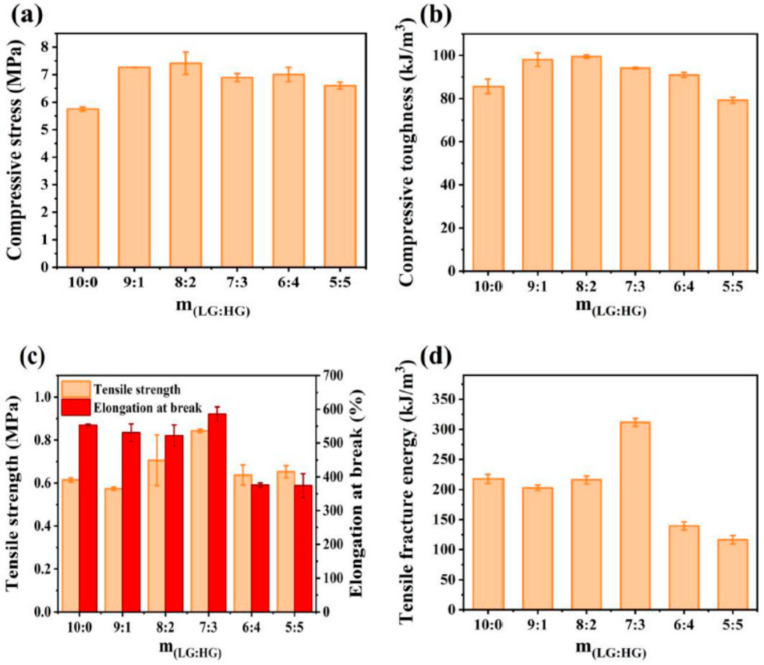
Influence of LG/HG weight ratio (m_(LG:HG)_) on (**a**) the compressive stress at 90% strain, (**b**) the compressive toughness, (**c**) the tensile strength and breakage elongation, (**d**) tensile toughness (10 wt.% solid content, 20% GG in PVA/GG mixture, 5 wt.% TA, 1 M Na_2_SO_4_). Adapted with permission from [202], copyright 2024, Elsevier Ltd.

PVA and gellan gum (GG) mixtures as biodegradable matrices in conductive hydrogels were prepared by applying the FT method, using in addition tannic acid and a treatment with Na_2_SO_4_ solution [202]. The double network (DN) structure with excellent mechanical properties was achieved when moderate GG content was added into the PVA matrix (it was found that the PVA/GG mixture with about 10% GG exhibits a synergistic behavior). This is due to the formation of hydrogen bonds between the –OH groups of PVA and GG as well as the hydroxyl and carbonyl groups of TA (the optimum content of TA was found to be 6% TA). Also, by applying three FT cycles, crystallites are formed by both PVA and GG chains. By freezing GG random coils, double helices are formed that further associate during thawing, the effect potentiated after immersion into Na_2_SO_4_ solution. The most important characteristics reported for this system were the following: a compressive toughness of 10.7 MPa (at 90% strain), tensile strength of 1.2 MPa, breakage elongation of 890%, fracture energy of 480 kJ/m^3^ and a conductivity value of 3.27 S/m. After 10 consecutive cycles under 200% strain, the stress recovery rate was about 86%. In the presence of LGG, the PVA-based hydrogel is hard but fragile, while HGG will lead to a flexible but soft network; a suitable proportion of LGG to HGG determines the optimum mechanical properties (Figure 15). The excellent mechanical properties are accompanied by low swelling in water (the equilibrium swelling ratio, ESR < 300%) and physiological saline solution (ESR < 90%) [202].

**Figure 16:**
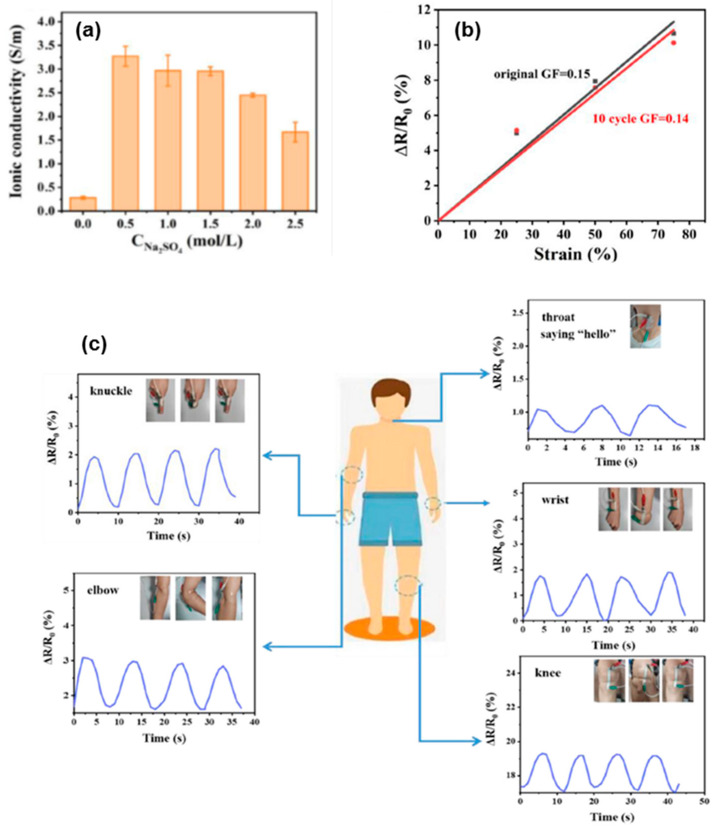
(**a**) The influence of Na_2_SO_4_ on the conductivity of the composite hydrogels (10 wt.% solid content, the weight ratios of 8:2 for m_(PVA/GG)_ and m_(LG:HG)_, 6 wt.% TA); (**b**) Comparison of GF for the sample without cyclic testing or after 10 consecutive tensile loading–unloading; (**c**) GG/PVA hydrogel (2 M Na_2_SO_4_) used for detecting the movement of different parts of the human body. Adapted with permission from [202], copyright 2024, Elsevier Ltd.

The performances shown for PVA/GG networks (mechanical properties, conductivity, strain sensing, low swelling and biodegradability), as well as the facile and ecofriendly preparation, make these hydrogels suitable for flexible electronics (Figure 16) [202].

**Figure 17:**
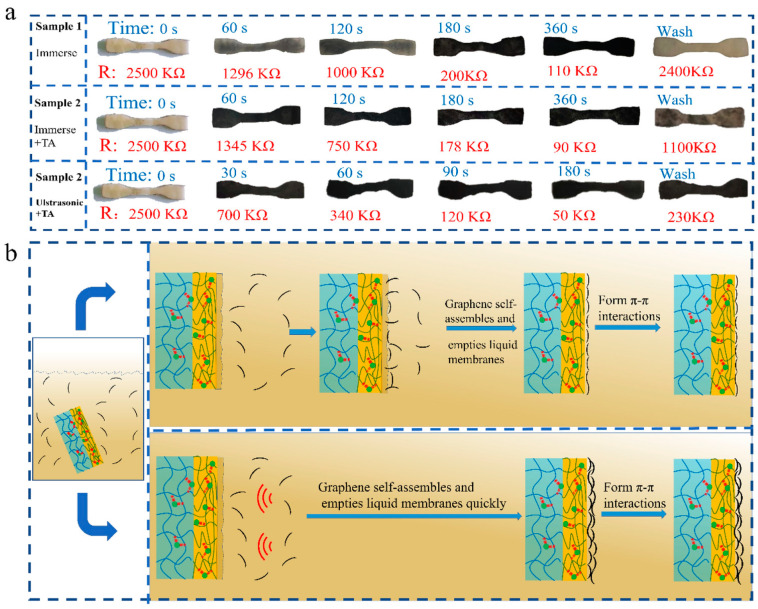
(**a**) Adhesion and resistance of graphene on hydrogel surface under different conditions. (**b**) Construction diagram of graphene conductive layer on water gel surface under stirring condition and ultrasonic assisted condition. With permission from [203], copyright 2024, Springer Nature.

**Figure 18:**
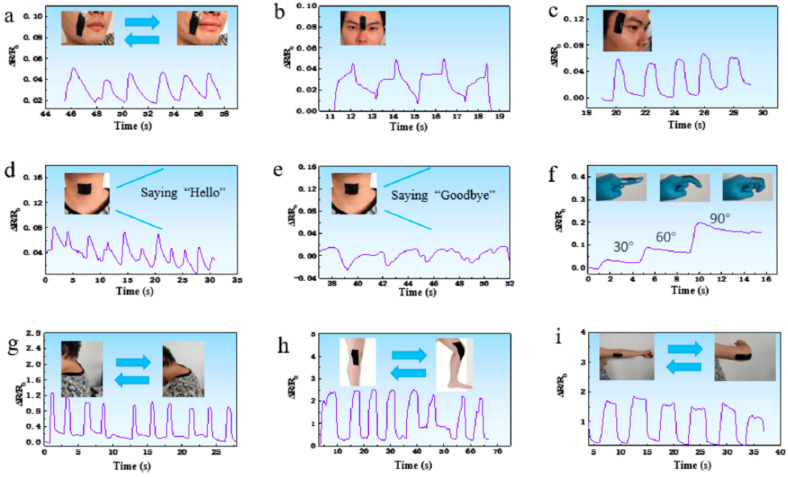
Relative resistance changes in strain sensor fabricated by PVA/TA/graphene hydrogel when used to monitor various human motions including (**a**) smiling, (**b**) frowning, (**c**) blinking, (**d**) saying “hello”, (**e**) saying “goodbye”, (**f**) finger bending, (**g**) head-down, (**h**) knee bending and (**i**) elbow bending. With permission from [203], copyright 2024, Springer Nature.

A sensor with improved sensitivity, durability over 1000 cycles and fast response (227 ms) was obtained by Liu et al. [203] consisting of a tight graphene conductive layer on the surface of the PVA/TA hydrogel (Figure 17), with a strain sensitivity of 0.1%. The H-bonds between PVA and TA and the strong π–π interactions between TA and graphene ensure the sensor performance during monitoring the human motions (Figure 18).

Remarkable performances were obtained by the combination of nanotube alignment with mussel-inspired chemistry [204]. Thus, the PVA matrices incorporating halloysite nanotubes, PDA and ferric ions (Fe^3+^) showed elongation up to 30,000% of the original length, maintained the electrical properties after 600% strain and displayed self-healing ability and strain sensitivity. Also, comparing these materials to their nonfunctional counterparts, they showed an increase of 3 times in electrical conductivity, 20 times in mechanical rigidity, and 7 times in energy dissipation. The multifunctional composites were 3D-printed for obtaining customized wearable devices for human motion sensing.

Smart biomimetic devices with high actuation strength value (above 200 kPa) were obtained with PVA in combination with natural rubber latex (NRL). A multiscale-oriented structure was obtained during stretch-drying PVA and NR, determining an enhancement in the mechanical strength (3.2 MPa) and shape memory (shape recovery ratio of ≈92%), able to operate at maximum efficiency when lifting a load 372 times its own weight [205]. It was recently shown that a small-waist structure enhances the sensitivity during the compression of the PVA hydrogel [206]. The ultrasonic dispersion technique was used to develop a biosensor with ultrahigh stretching, biocompatibility, antibacterial activity and wound healing ability. A multiple crosslinking network was obtained from PVA, cellulose, sodium alginate (SA) and zinc oxide and was able to detect sound vibration and joint motion [207].

### 4.4. Other New and Promising Applications of PVA-Based Hydrogels

**Figure 19:**
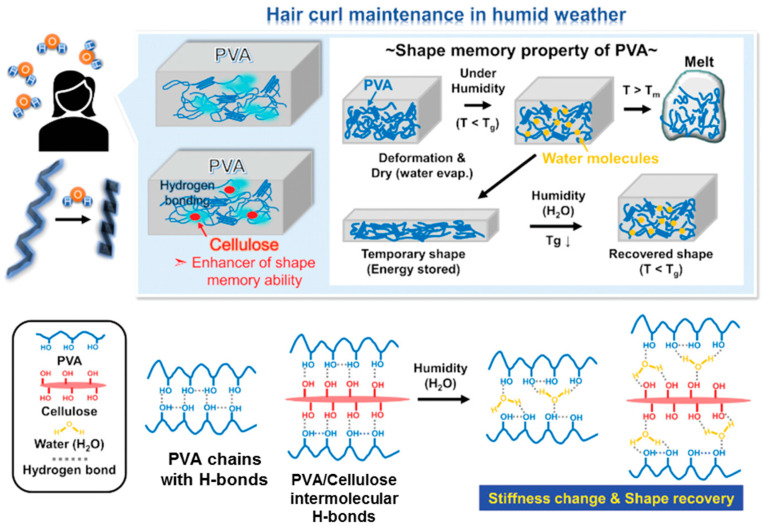
Schematic illustration of humidity-responsive shape memory mechanism of PVA/MC composites and its application in hair styling. H-bonds among PVA, cellulose and water molecules are also shown. Adapted with permission from [208], copyright 2023, Wiley-VCH GmbH.

**Figure 20:**
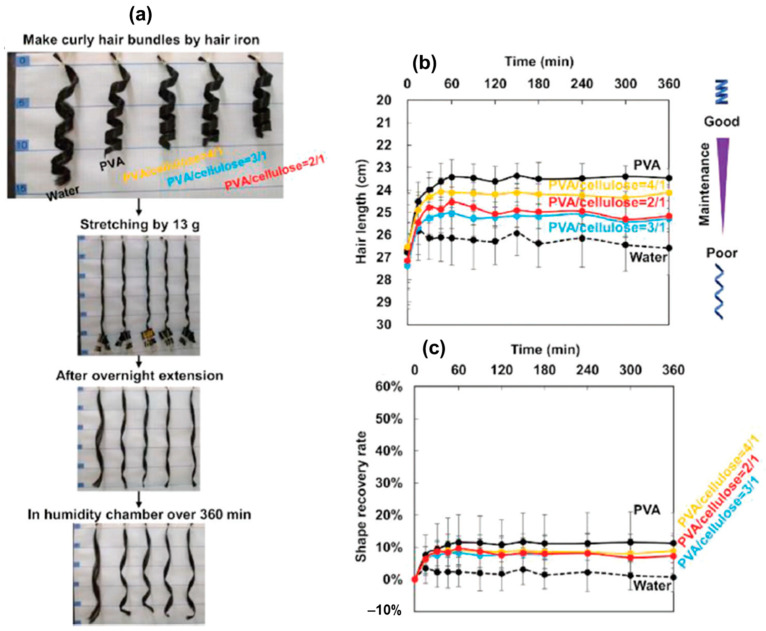
(**a**) Photographs of humidity-responsive shape fixation and shape recovery process of curly hair bundles coated with PVA and PVA/MC composites. Time curves of (**b**) length and (**c**) shape recovery rate of temporarily stretched curly hair bundles when exposed to 80% relative humidity. Adapted with permission from [208], copyright 2023, Wiley-VCH GmbH.

Customized hair styling is an important issue for a modern lifestyle. In muggy and humid weather, moisture penetrates the hair, leading to a frizzy or reduced volume appearance. It was recently shown that the use of PVA and microcrystalline cellulose (MC) ensures hair curls maintenance in humid environments [208]. The composite films were prepared from aqueous solutions of PVA/MC mixtures. For 20–25 wt.% cellulose in the polymer mixture, the maximum recovery was achieved. In aqueous solution, the –OH groups from the PVA and cellulose chains developed H-bonds (Figure 19). When the curly hair bundles were coated with PVA and PVA/MC solutions, the stiffness change and shape fixation were improved, maintaining the shape of curled hair bundles for at least 6 h, at ≈30 °C, under 80% humidity and promoting the hair shape recovery process with a rate of about 8–10%. The PVA/MC composites in different weight ratios (4/1, 3/1 and 2/1) were tested to tailor the PVA-MC filler interactions in order to enhance the humidity-responsive shape fixation and shape recovery process of curly hair bundles (Figure 20) [208].

Composite hydrogels of cellulose-reinforced PVA determine a decrease in pore size, while their number increased as compared with pure PVA hydrogel. Such a hydrogel was tested in cosmetic formulations, as vehicle for the niacinamide release via the transdermal route [209]. Electrospun PVA membranes containing a high amount of TA were used as filter layers for face masks with antibacterial properties, excellent UV-shielding and particle filtration efficiency [210].

Semiconducting polymers, such as polyaniline (PANI), were included in the PVA porous matrix to design conductive composites as flexible materials for supercapacitors with high cyclic stability (~95% after 1000 cycles) [211] and conductivity (up to 0.29 S/cm [212]) and improved mechanical properties [212,213], suitable as macroporous scaffolds for tissue engineering or membranes for bioseparation. Double-layer electrode/electrolyte hydrogels with self-healing ability were prepared by introducing poly(2-acrylamido-2-methyl-1-propane) sulfonic acid doped with PANI [214]. It was suggested that these flexible materials can be used to produce wearable energy storage devices.

Also, the synergistic strategies concerning the gelation induction in the presence of monocarboxylic acid or the freezing/thawing method and salting-out effect was adopted to produce strong and tough conductive PVA hydrogels for flexible solid-state supercapacitors [34]. Acetic acid addition to DMF solutions or the application of freezing/thawing cycles triggers the formation of PVA crystalline domains resulting in a physical network. This is then soaked into salt solutions of suitable concentrations until the solvent exchange equilibrium is reached (approx. 24 h), modulating the non-covalent interactions (hydrogen bonding or hydrophobic interactions), enhancing mechanical properties and ionic conductivity of the network. Such tough and conductive hydrogels are suitable for flexible energy storage devices [34,38].

Another recent study demonstrated that the addition of lithium iodide reduces the hydrogen bonds between the molecular chains of PVA, improves the molecular orientation of macromolecular chains and decreases the crystallinity, resulting in the enhanced spinnability and mechanical strength of PVA fibers [215]. Polymer gel electrolytes present high interest as carriers for cation and anion transport. The PVA matrix incorporating TEOS was tested as a gel for valve-regulated lead acid battery application [216].

In an aqueous two-phase system in a moderate basic environment, a layer-by-layer 3D-printed physically crosslinked network was generated using PVA samples with the polymerization degree (*n*) between 500 and 1700 dissolved in solution of salt mixture (Na_2_SO_4_ and NaOH) [35]. The viscosity of PVA inks with concentrations in the range of 5% to 25% (*w*/*w*) was between 0.1 Pa s and 20 Pa s.

Porous membranes can be obtained by modulating a thermodynamic parameter (e.g., solubility parameter, temperature) that induces a phase separation into PVA-rich and PVA-poor phases. After demixing occurrence, the morphology and porosity required consolidated either by gelation [217] or crystallization/vitrification of the polymer rich phase [218].

PVA-based hydrogels represent promising soft adhesive materials for various applications. PVA/GO hydrogels obtained in water/DMSO mixture have shown good mechanical properties and strong adhesion in air water or oil (adhesion strength on aluminum, copper plastic and glass was between 50 and 150 kPa) [219]. High underwater adhesive strength was also observed using PVA/TA hydrogels on various materials (metals, polymers, ceramics etc.) [220]. Using the multiple network approach, PVA/BSA hydrogels with adjustable mechanical viscoelasticity were developed using the FT method combined with BSA in the presence of genipin and reduced glutathione [55]. They provided self-healing ability, shape integrity and improved bioadhesion, these hybrid materials being suitable as candidates for tissue repair and regeneration. Another hydrogel consisting of a PVA/chitosan composite with good biocompatibility, anti-inflammatory effects and wet tissue adhesion properties was obtained by crosslinking with genipin and tested as a biomaterial for preventing postoperative abdominal adhesion [221].

Another important application of PVA-based hydrogels is represented by materials used for environmental protection. PVA/SA DN hydrogels were prepared by FT and La(III) ionic crosslinking [222]. The combined effects of electrostatic and Lewis acid/base interactions, as well as the ligand/anion exchange, determine the efficient phosphate adsorption over wide pH values, from 3 to 12. Another DN approach used PVA/SA and polyhexamethylene biguanide hydrochloride to prepare efficient marine antifouling materials [223]. A laccase mediator system, obtained by the in situ synthesis of MOF in the presence of PVA, Zn^2+^ and Cu^2+^ via the FT method, was found as an efficient material for wastewater treatment [68,224].

PVA/lignin hydrogel prepared by the FT and chemical crosslinking combined methods was recently proposed as an effective adsorption material for wastewater treatment, displaying an adsorption efficiency of 193.8 mg/g and 190.0 mg/g for methylene blue and crystal violet, respectively [225]. PVA blends with quaternized polysulfones with optimal composition allow for the modulation of membrane properties [226]. By coating a polysulfone substrate with a PVA/TA hydrogel, the functionality of the membrane was improved against dye/salt mixtures (approx. 99% Congo Red rejection) [227].

A hydrogel of PVA, gallic acid and lysozyme was developed as a packaging material with potential uses in food preservation [228]. The hydrogel presented thermal stability, antibacterial properties (against *Escherichia coli* and *Staphylococcus aureus*), complete UV blocking ability, a fracture strength of ~37.9 MPa, an elongation at break of ~40% and water vapor and O_2_ barrier properties, extending the shelf life of blueberries to about 20 days. Another stretchable hydrogel, composed of PVA, sodium carboxymethyl cellulose, poly(ethylene imine) (PEI) and TA [229], presented good performances, including elongation ~400%, self-healing ability, full UV blocking (<400 nm) and an adhesive strength of 234 KPa. This composite demonstrated efficiency in fruit preservation (prolonging the shelf life by at least one week for strawberries, mangoes and cherries) [229]. Tough and flexible PVA/xanthan gum composite cryogels loaded with polyphenol extracts from red grape pomace were also proposed for food packaging applications [230].

**Scheme 2:**
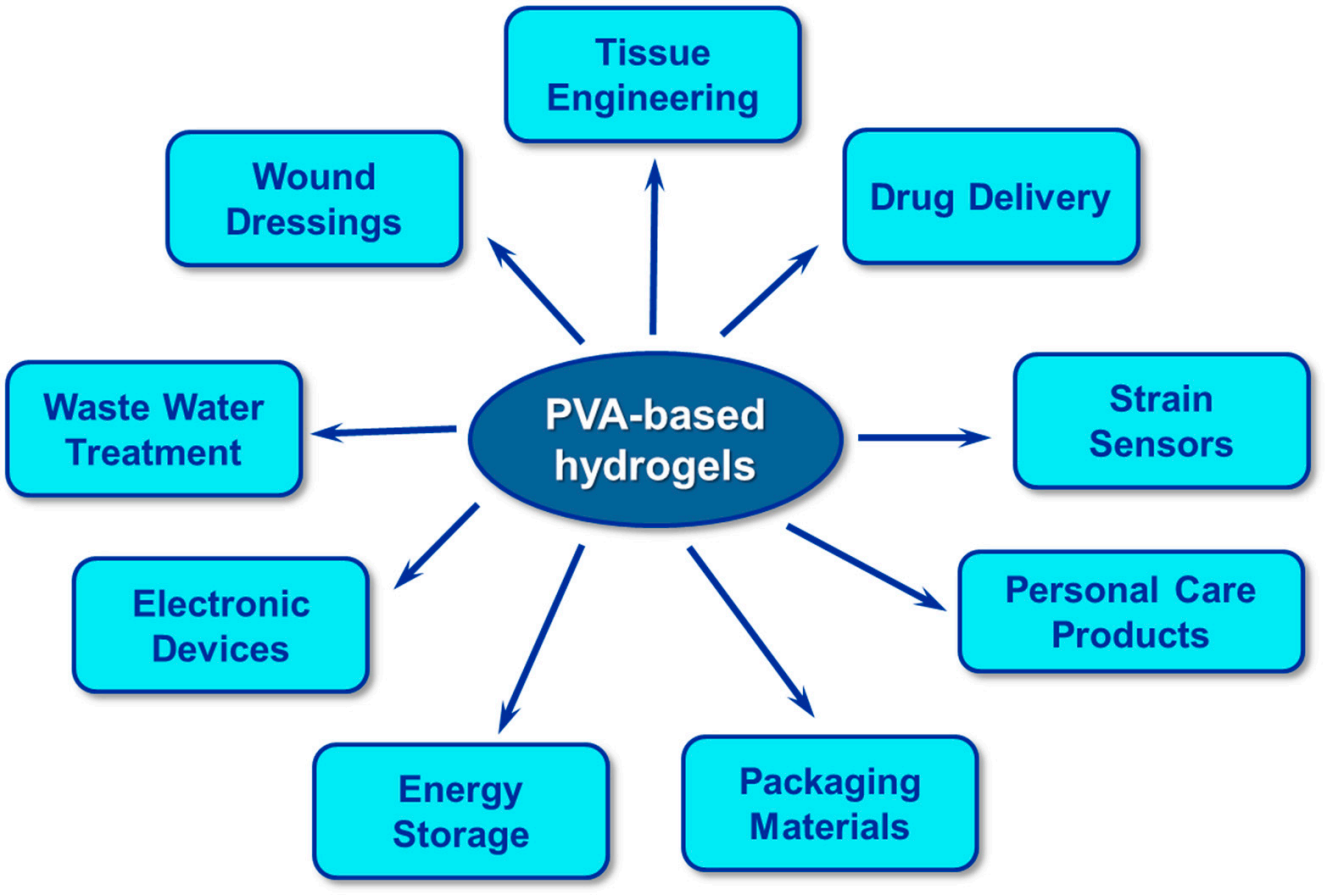
The most important applications of PVA-based hydrogels.

In conclusion, PVA-based hydrogels can be regarded as promising innovative and adaptable materials for a wide range of uses (Scheme 2).

## Figures and Tables

**Table 1:** The main techniques used for the characterization of PVA hydrogels.

Method	Acquired Information
Scanning Electron Microscopy (SEM)	– morphology (porosity, pore distribution) – composition of hydrogels
Mechanical Properties	– mechanical strength and toughness – hardness and wear
Rheology	– viscoelastic properties of hydrogels – network strength and stiffness – crosslinking density – shear thinning – yield stress – thixotropy (self-healing ability)
Fourier-Transform Infrared (FTIR) Spectroscopy	– structural arrangement in hydrogel by comparison with starting materials – intermolecular interactions (hydrogen bonds, hydrophobic interactions)
Thermo-Gravimetric Analysis (TGA)	– changes in material properties as function of temperature – thermal degradation profile
Differential Scanning Calorimetry (DSC)	thermal transition behavior and associated enthalpy for each process – glass transition temperature – melting temperature – crystallization temperature
Dynamic Mechanical Analysis (DMA)	– viscoelastic properties in solid state under action of small cyclic deformations
Small-Angle Neutron Scattering (SANS)	– anisotropy at small length scales
Atomic Force Microscopy	– surface topography
Swelling Behavior	– water and fluid absorption into network
X-Ray Diffraction	– degree of crystallinity
Sol–Gel Analysis	– yield of crosslinking and degradation – gelation dose
Contact Angle Analysis	– hydrophilicity of materials
Biocompatible Tests	– cytotoxicity – thrombogenicity – bioadhesion

## Abbreviations

CNF	cellulose nanofibril
DH	degree of hydrolysis
DN	double network
ESR	equilibrium swelling ratio
FT	freezing/thawing
GA	glutaraldehyde
GF	gauge factor
GG	gellan gum
HGG	high-acyl gellan gum
LGG	low-acyl gellan gum
GO	graphene oxide
GMA	glycidyl methacrylate
H-bond	hydrogen bonding
MC	microcrystalline cellulose
*n*	polymerization degree
PA	phytic acid
PVA	poly(vinyl alcohol)
PAm	polyacrylamide
PANI	polyaniline
PDA	polydopamine
SA	sodium alginate
SFT	suppressed freezing/thawing
TA	tannic acid
TEOS	tetraethyl orthosilicate
ZIF-8	zeolitic imidazolate framework
